# SMAP: a streamlined methylation analysis pipeline for bisulfite sequencing

**DOI:** 10.1186/s13742-015-0070-9

**Published:** 2015-07-01

**Authors:** Shengjie Gao, Dan Zou, Likai Mao, Quan Zhou, Wenlong Jia, Yi Huang, Shancen Zhao, Gang Chen, Song Wu, Dongdong, Li, Fei Xia, Huafeng Chen, Maoshan Chen, Torben F Ørntoft, Lars Bolund, Karina D Sørensen

**Affiliations:** 1 Department of Molecular Medicine, Aarhus University Hospital, Aarhus, Denmark; 2 BGI Co Ltd, Shenzhen, 518083 China; 3 School of Computer Science, National University of Defense Technology, Changsha, 410073 China; 4 Shenzhen Second People’s Hospital, the First Affiliated Hospital of Shenzhen University, Shenzhen, China; 5 Department of Biomedicine, Aarhus University, Aarhus, Denmark; 6 Department of Computer Science, City University of Hong Kong, Hong Kong, 999077 China; 7 Electronic Engineering College, Naval Engineering University, Jiefang Avenue #717, Wuhan, 430033 China; 8 Genomic Biology Laboratory, QIMR Berghofer Medical Research Institute, Brisbane, Australia

**Keywords:** Reduced representation bisulfite sequencing (RRBS), Differentially methylated region (DMR), Allele-specific DNA methylation (ASM)

## Abstract

**Background:**

DNA methylation has important roles in the regulation of gene expression and cellular specification. Reduced representation bisulfite sequencing (RRBS) has prevailed in methylation studies due to its cost-effectiveness and single-base resolution. The rapid accumulation of RRBS data demands well designed analytical tools.

**Findings:**

To streamline the data processing of DNA methylation from multiple RRBS samples, we present a flexible pipeline named SMAP, whose features include: (i) handling of single—and/or paired-end diverse bisulfite sequencing data with reduced false-positive rates in differentially methylated regions; (ii) detection of allele-specific methylation events with improved algorithms; (iii) a built-in pipeline for detection of novel single nucleotide polymorphisms (SNPs); (iv) support of multiple user-defined restriction enzymes; (v) conduction of all methylation analyses in a single-step operation when well configured.

**Conclusions:**

Simulation and experimental data validated the high accuracy of SMAP for SNP detection and methylation identification. Most analyses required in methylation studies (such as estimation of methylation levels, differentially methylated cytosine groups, and allele-specific methylation regions) can be executed readily with SMAP. All raw data from diverse samples could be processed in parallel and ‘packetized’ streams. A simple user guide to the methylation applications is also provided.

**Electronic supplementary material:**

The online version of this article (doi:10.1186/s13742-015-0070-9) contains supplementary material, which is available to authorized users.

## Findings

### Introduction

As an epigenetic marker in mammalian cells, DNA methylation (methylation of the DNA base cytosine, C, to form 5-methylcytosine) affects genetic imprinting and cellular specification without altering DNA sequences [1, 2]. In mammalian cells, most methylation occurs at CpG dinucleotides. Most of the CpG dinucleotides are methylated, but non-CpG methylation frequently occurs in the brain cells and embryonic stem cells of mammals [3, 4]. DNA methylation also regulates gene expression. In promoter regions, unmethylated CpGs usually activate transcription by binding of specific transcription factors, whereas methylated CpGs can ‘silence’ transcription by preventing binding [5]. Furthermore, dysregulation of DNA methylation is a hallmark of cancer. Genomic demethylation and gene-specific hypermethylation occur most notably in oncogenes and tumor-suppressor genes, respectively [6]. DNA methylation has also been used as a biochemical predictor for cancer recurrence [7].

Whole-genome bisulfite sequencing (Bis-seq) has been developed to detect methylation [8–10]. Treatment of DNA with sodium bisulfite converts cytosine residues into uracil, but 5-methylcytosine residues are unaffected. Thus, methylated and unmethylated CpG sites can be discriminated [11]. Although the price of next-generation sequencing has been decreasing, ensuring that Bis-seq is affordable for most laboratories will take some time. Furthermore, uneven distribution of methylated cytosine residues in double strands in the genome makes it difficult for Bis-seq to detect differentially methylated regions and single nucleotide polymorphisms (SNPs), especially in low-coverage regions [12]. Reduced representation bisulfite sequencing (RRBS) is a cost-efficient and high-throughput method to analyze methylation profiles with the resolution of a single nucleotide [8]. By increasing sequencing depth in target regions, RRBS also easily tackles the problem of uneven distribution. Development of RRBS technology has increased the demand for well designed bioinformatics tools to facilitate subsequent data analyses.

Various tools have been developed for methylation calling and/or further analyses of RRBS data [13] (Additional file 1: Table S1). Differential methylation analysis package (DMAP), Methylkit and methylSig perform differential methylation analyses, with the latter using a beta-binomial approach to account for read coverage and biological variation [14–16]. Bis-SNP [12] is the only widely used package capable of RRBS data-based SNP detection, which is important for identification of allele-specific epigenetic events such as allele-specific methylation (ASM) and imprinting [17–19]. Amrfinder (now a part of the MethPipe package) presents a statistical model to describe ASM [20, 21]. Several mapping applications have also been developed and used in Bis-seq or RRBS data processing, and will be discussed below.

Despite the existence of these tools, many questions remain. First and foremost, paired-end (PE) reads with overlapping regions represent duplicated information. Without removing such redundancy, estimation of the methylation rate could be biased considerably. Counting sites in overlapping regions for PE sequencing only once would fully recover the correct methylation rate and hence greatly reduce errors in the subsequent calculation of differentially methylated regions (DMRs) and differentially methylated C sites (DMCs). However, few currently available RRBS analysis applications account for such duplication. Amrfinder includes a novel statistical model to detect ASM [21]; however, its prerequisite of equal allele frequency is not suitable for more complex cancer cases.

Here, we present a streamlined package called SMAP to meet the need of extracting multiple types of information (such as DMCs, DMRs, SNPs and ASM) from various types of RRBS and Bis-seq data.

### Pipeline

**Fig. 1:**
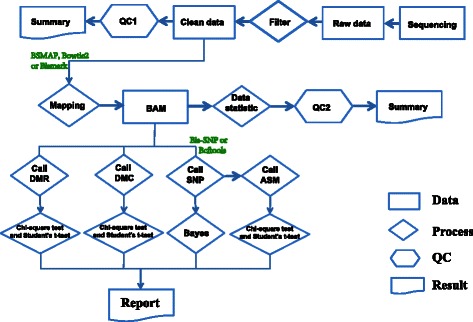
Brief workflow of SMAP. SMAP filters sequenced raw data and produces ‘clean data’. Clean data is then mapped onto the pretreated reference genome. Finally, SNPs, ASM, DMRs and DMCs are detected and reports created. Existing tools are in green

SMAP is a modular pipeline implemented in Perl that calls software components written in C/C++, Perl, R and Java. Required input files for SMAP include Bis-seq or RRBS data in FASTQ format, and a user-defined configuration file that includes all settings of the pipeline (a pipeline document and full example is given in Additional file 2). SMAP can resume broken runs and aims at comprehensive and convenient processing of Bis-seq and RRBS data (Fig. 1, Additional file 1: Table S1). The pipeline consists of seven operational stages: (i) reference preparation, (ii) read preparation, (iii) alignment, (iv) calculation of methylation rate, (v) DMR detection, (vi) SNP and ASM calling and (vii) summarization (Fig. 1). Details of workflow are described below.

#### Step 1. Reference preparation

All Cs in the reference genome sequence are converted into Ts for both strands. Original and converted double-strand reference sequences are then indexed by Bowtie2. User-defined RRBS restriction enzymes (multiple restriction enzymes are supported) are used to digest the references into 40–220 bp segments (subsequently called ‘target regions’).

#### Step 2. Read preparation

A script was developed to remove adaptors and low-quality regions in raw RRBS reads. Different from other popular pipelines for filtering of raw reads, this script does not simply remove the whole reads including adaptors. Instead, it ‘trims’ the ends with adaptors off the reads, which maximizes the amount of clean data.

#### Step 3. Alignment

One of the three representative mapping tools, Bowtie2, Bismark or BSMAP, is used for mapping reads onto reference genomes in SMAP. Users decide which to use. BSMAP is a wildcard-based application, whereas Bowtie2 and Bismark are often used to map reads onto the three-base references prepared in step 1.

#### Step 4. Calculation of methylation rate

Taking account of PE read overlap (see below), the rate of methylation is calculated for each C in target regions. Rates are used for later analyses.

#### Step 5. Detection of DMCs and DMRs

A core region with a certain number (default 5) of CpG dinucleotides is used as the seed to call differentially methylated regions between two samples, such as ‘cancer’ and ‘normal’ samples. Then, the seed is prolonged by checking CpG dinucleotides one-by-one according to the results of the *t*-test and chi-square test of the difference in methylation rates between samples. When the number of reads is <5, the chi-square test is replaced by Fisher’s test automatically. A change in the trend of the methylation rate stops the elongation. Fully elongated regions are defined as DMRs. Similar tests are applied to each C to detect DMCs.

#### Step 6. Detection of SNPs and ASM

The current version of SMAP uses Bis-SNP or Bcftools to call SNPs, depending on the mapping tool selected by the user. That is, when BSMAP or Bismark is selected, Bis-SNP is called, whereas if Bowtie2 is selected Bcftools is called. We are also developing a novel SNP calling algorithm that shows a 100-fold faster speed and comparable accuracy (unpublished data) and which will be integrated into SMAP to replace the Bcftools pipeline. Heterozygous SNPs are chosen to detect ASM events (see below).

#### Step 7. Summarization

Mapping and coverage information is tabulated in a final report. See Section 4.1 in Additional file 2 for an example that includes one para-normal sample (normal tissue adjacent to cancer tissue at ≥5 cm; called ‘normal’ later) and three cancer samples (primary renal cell carcinomas (pRCCs), local invasion of the vena cava (IVC) and distant metastasis to the brain (MB) tissues from a patient with metastatic renal cell carcinoma [22]). In addition, this dataset also includes exome sequencing data used for validation of the performance of the pipeline (see below).

As a part of quality control (QC), the coverage of CpG islands by target regions is also plotted to assess the quality of sequencing data. RRBS sequencing regions covers about ≈ 57 % of CpG islands when the length of target regions is 40–220 bp. The number of sites covered by RRBS data decreases with increasing number of supporting reads. If the amount of sequenced data is sufficient and the quality of data is good, the decrease slows with increasing number of supporting reads. In the example given above, as the coverage of 1X, 4X, 10X and 20X supporting reads decreases slowly, this QC is passed. In particular, 10X sequenced reads in CpG islands covered about ≈ 80 % (44/57) of target regions for normal tissue (see Additional file 1 Figure S1, S2, S3 and S4). This finding demonstrates a high-quality sequencing dataset. An example with a configuration file and main output is shown in Additional file 2.

### Comparison of the performance of alignment tools

To determine the locations of methylated sites and SNPs, sequencing reads must first be mapped onto the corresponding reference genome. Several types of alignment software have been developed to map bisulfite converted reads [23]. These types of software can be classified into two groups. The first group (e.g., BSMAP [24] and RRBSMAP [25]) is based on mapping of raw data and uses wildcard alignment. The second group (e.g., Bowtie2 [26], SOAP [27, 28], MAQ [29], Bismark [30], BRAT-bw [31] and BS Seeker [32]) needs additional C → T conversion preprocessing and complex post-processing but leads to higher accuracy. Bismark and BS Seeker are based largely on Bowtie. As representatives of these methods, BSMAP, Bismark and Bowtie2 are currently used in SMAP. Bowtie2 and Bcftools are not specially designed for Bis-seq data, so we developed programs to integrate them seamlessly into SMAP.

Simulated data were used to evaluate the performance of alignment in various conditions. First, we created (*in silico*) enzyme MspI-cleaved DNA segments with a length of 90–220 bp distributed randomly on chromosome 18 (chr18) in the GRCh37 assembly (hg19) of the human genome. Around 0.05 % of sites were selected randomly as SNPs on the segments. Paired-end reads with length of 50, 60, 70, 80 and 90 bp were then simulated on both Watson and Crick strands of these segments with even distribution and 10X coverage for cancer tissue and normal tissue. BSMAP and Bismark were used to map the reads onto the reference genome. Unsurprisingly, with increasing read length, mapping rate and accuracy increased and false-positive rates decreased. The mapping rate of Bismark is lower than that of BSMAP, which was also shown in its higher false-negative rate. False-negative rates of BSMAP are zero for all types of reads. Most reads that failed to map onto the reference were present in repeated regions. Despite the lower mapping rate, the accuracy of Bismark was higher, especially for 50 bp and 60 bp reads, though its absolute number of accurately mapped reads was less than that of BSMAP for longer reads (those with length of 80 bp and 90 bp; Additional file 1: Table S2).

### Overlap treatment for PE data and detection of methylation

**Fig. 2:**
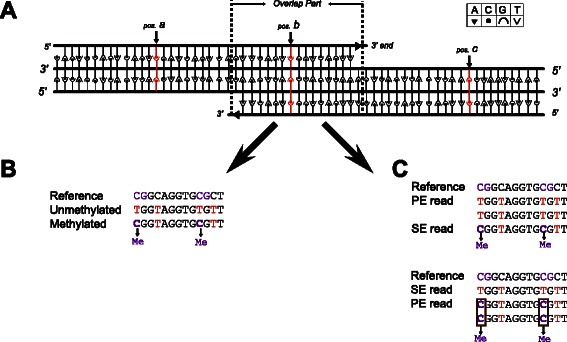
Effects of bias correction in overlapping regions of PE reads (schematic). Me: Methylated site. **a** The overlapping region of a pair of PE reads. The C bases in position a and c are not in overlapping regions. However, the C base in position b is in an overlapping region of the pair of reads. **b** A case assuming methylated reads have the same proportion as unmethylated reads (i.e., methylation rate is 50 %). **c** The upper panel shows a case assuming that unmethylated samples were sequenced by PE sequencing and methylated samples were sequenced by SE sequencing. The lower panel shows a case in which unmethylated samples were sequenced by SE sequencing whereas methylated samples were sequenced by PE sequencing

In RRBS analyses, no currently available software takes into account the overlap of PE reads, which can cause considerable bias in DMR detection. As a simple example, three CpG sites exist in a target fragment and two PE reads are overlapping. One methylated CpG site is located in the region where the reads overlap. The other two CpG sites are not methylated and exist in non-overlapping regions. Thus, the ‘true’ methylation rate of this fragment is 33 % (Fig. 2a). However, if overlapping treatment is not taken into account, the methylation rate of this region becomes 50 %. In another example, we assume the same proportion of methylated and unmethylated reads (i.e., methylation rate is 50 % (Fig. 2b)). If unmethylated sequences are sequenced by PE sequencing and methylated samples are sequenced by SE sequencing, and if overlapped PE sites are counted twice, then the methylation rate becomes 33 %, smaller than the true value (left panel of Fig. 2c). If unmethylated samples are subject to SE sequencing whereas methylated samples are subject to PE sequencing, the methylation rate becomes 66 %, larger than the true rate (right panel of Fig. 2c). Thus, lack of bias correction could increase or decrease estimation of methylation rates.

To correct such bias, we count sites in overlapping regions for PE sequencing only once. This treatment fully recovers correct methylation rates and hence greatly reduces errors in subsequent calculation of DMRs. For example, in a certain genomic region, actual methylation rates in normal tissue and cancer tissues are 50 % (i.e., this region is not a DMR if our redundancy-removing strategy is used). However, if overlapping PE sites are counted twice, the region might be falsely annotated as a DMR. Our strategy decreases the false-positive rate.

To assess the performance of methylation detection, 10 large segments (length range, 1.5–13.6 Mbp) on chr18 with various methylation rates were selected from the simulation described above, and methylated sites were also simulated. To simulate methylated sites, the methylation rate for each segment was assigned randomly. Based on these theoretical rates, methylated sites were designed to be distributed randomly on segments. Methylated sites were then estimated based on the alignments mentioned above for each segment using SMAP. Estimated methylation rates were highly consistent with simulated (theoretical) values regardless of read length, tissue type or mapping method (Additional file 1: Table S3). Considering the high mapping rate shown above, methylation detection was shown to perform well in SMAP.

### DMR detection and SMAP performance

Detection of differentially methylated regions has a critical part in study of the mechanism, recurrence, diagnosis and treatment of cancer [7, 33–36]. Based on single-C methylation information of patient and control samples, Pearson’s chi-square test is used to ascertain whether methylation rates of concerned regions in different samples are sufficiently different to be identified as DMRs. The region is defined as a DMR if p < 0.05 (chi-square test) and the difference in methylation rates between cancer tissue and normal tissue is >0.1. We randomly selected 12 DMRs detected by bisulfite-sequencing PCR [22]. All of them were confirmed by SMAP, which illustrates the high accuracy of our method (Additional file 1: Table S4).

### Comparison of SNP detection pipelines

Bis-SNP is the most popular SNP-detection software for RRBS data. In SMAP, it is used with mapping tools BSMAP and Bismark. Bowtie2 output is not compatible with Bis-SNP. Bismark undertakes format conversion and makes it compatible. To make SMAP more flexible, a pipeline using conventional tools was also developed. Bcftools is used to call SNPs using Bowtie2 alignments. Here, the performance of SNP-calling pipelines was evaluated by comparison of estimated SNPs using data from exome sequencing or RRBS from the four samples mentioned above. BSMAP and Bowtie2 pipelines illustrated a higher SNP call rate (≈70 %) than the Bismark pipeline (≈40 %). However, the Bismark pipeline showed much lower false-positive rates in all tissues (Additional file 1: Table S5). The overlap of correctly estimated SNPs was high between samples (Additional file 1: Figure S5a) as well as between BSMAP and other pipelines (Additional file 1: Figure S5b). However, some SNPs by the Bowtie2 pipeline were shared only by the BSMAP pipeline (Additional file 1: Figure S5b). SNP-calling performance was assessed further by the simulated PE data with 50 and 90 bp reads mentioned above. BSMAP and Bismark pipelines showed considerable overlap in terms of correctly estimated SNPs. The Bowtie2 pipeline again shared fewer estimated SNPs with other pipelines, probably because the Bowtie2 pipeline called more homozygous SNPs whereas the other two pipelines were good at calling heterozygous SNPs (Additional file 1: Figure S6). For 50 bp read PE data, the Bismark pipeline was better than the other pipelines with regard to the number of SNPs called, whereas the BSMAP pipeline performed best for 90 bp reads (Additional file 1: Figure S6).

**Fig. 3:**
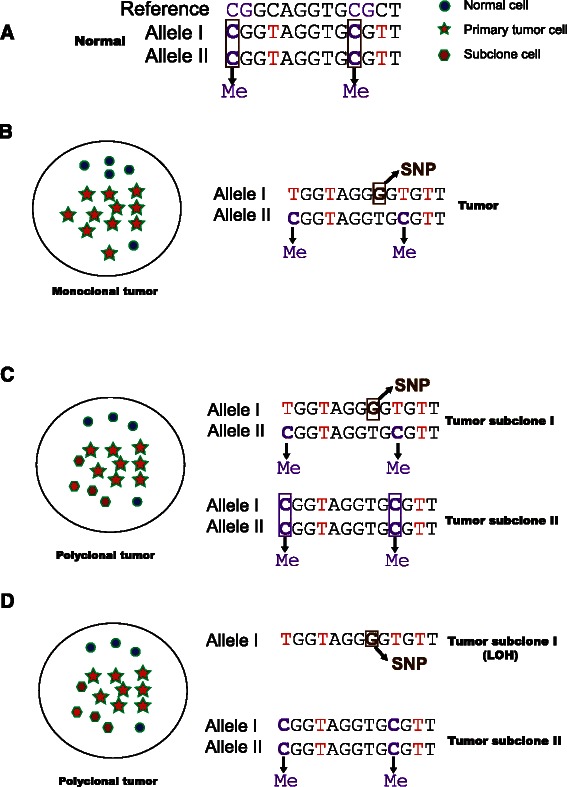
ASM detection. Purple Cs are methylated, whereas red Ts are not methylated. Me: Methylated site. **a** Basic case in which two C bases are methylated. **b** An example of an ASM region in a monoclonal tumor marked by a heterozygous G/T SNP. **c** An example of a polyclonal tumor in which the heterozygous SNP and reference allele are present. **d** An example of another type of polyclonal tumor in which the heterozygous SNP was changed to a homozygous G allele due to loss of heterozygosity

### A novel pipeline for ASM detection

Amrfinder is a popular ASM detect tool in which a statistical model is implemented to detect ASM [21]. However, the model applies only to the simple case of monoclonal cells with equal allele frequencies, which is not suitable for more complex cancer cases. In SMAP, heterozygous SNPs are used to determine alleles in two strands. To clarify this concept, an example is shown in Fig. 3. We assume there are no SNPs in normal cells (Fig. 3a). In the second situation, the monoclonal tumor (Fig. 3b), one T → G somatic SNP is present in allele I. In the third situation, the first type of polyclonal tumor (Fig. 3c), not only the T → G SNP (subclone 1) but also the original reference allele (subclone 2) is present. This SNP is the marker used to define the allele in the second and third cases. In the fourth situation, the second type of polyclonal tumor (Fig. 3d), the T/G heterozygous SNP in subclone 1 is changed to the homozygous G allele, whereas the reference allele T in subclone 2 is still present. The change is the result of loss of heterozygosity. The SNP G/T is also the marker of the allele. Once the allele is defined, the chi-square test is used to examine the significance of the difference of methylation rates in the two alleles. An ASM event is defined if (i) a heterozygous SNP locates on the same read as the relevant CpG (linked SNP-CpG pair), (2) p < 0.05 (chi-square test) in the read, and (iii) difference in methylation rates is >0.1.

Performance of ASM detection was assessed by simulated data. Ten segments were selected on chr18. Methylation rates of 0 to 1 were assigned to segments. Three segments were assigned a methylation rate of 0.5. All heterozygous SNPs with CpGs on same reads (with length of 50 bp or 90 bp) in these three segments were linked at least one ASM event. SMAP then estimated ASM events using Bowtie2, BSMAP and Bismark pipelines from simulated data. For 50 bp read PE data, BSMAP and Bismark pipelines performed similarly, whereas the Bowtie2 pipeline showed a high false-negative rate due to its disadvantage of calling heterozygous SNPs. For 90 bp read PE data, Bismark and Bowtie2 pipelines showed higher accuracy whereas BSMAP pipelines illustrated better sensitivity (Table 1). Interestingly, all SNPs not validated for ASM events were real SNPs but located in the seven segments without simulation of ASM events.

**Table 1:** Assessment of the performance of ASM

	PE90			PE50		
	BSMAP	Bismark	Bowtie2	BSMAP	Bismark	Bowtie2
Number of simulated ASM SNPs	158			108		
Number of simulated ASM events	1022			473		
Number of estimated ASM SNPs	136	93	55	72	79	30
Number of estimated ASM events	995	726	254	355	386	74
Number of validated estimated ASM SNPs	125	89	51	64	72	29
Number of validated estimated ASM events	843	719	239	338	372	73
FPR (ASM events)	0.15	0.01	0.06	0.05	0.04	0.01
FNR (ASM events)	0.18	0.3	0.77	0.29	0.21	0.85

Abbreviations: *ASM* allele-specific, *DNA* methylation, *FNR* false negative rate, *FPR* false positive rate, *PE50* 50 bp read PE data, *PE90* 90 bp read PE data, *SNP* single nucleotide polymorphism

## Discussion

Methylation studies have entered the era of single-base resolution since the advent of Bis-seq. RRBS technology allows targeting of CpG-rich regions and greatly reduces the cost of sequencing. It also promotes the creation and improvement of related bioinformatic analyses. Computational identification of DMRs, SNPs and ASM from RRBS data is a rapidly developing field, as illustrated by two DMR analytical applications published this year [14, 15]. At present, Bis-SNP [12] is the first and most widely used SNP-identification algorithm for RRBS data. None of the currently available methylation-analysis tools correct for PE overlap bias, and no suitable ASM pipeline is available for complex cancer data. Furthermore, some of the methylation tools are not convenient to use. To address these problems, we developed SMAP, which is designed to be an easy-to-use, one-stop and sophisticated package for methylation analyses. Some features of SMAP and comparisons with other types of software are shown in Additional file 1: Table S1. SMAP showed good performance in most cases.

In our previous work on a case of metastatic renal cell carcinoma [22], we undertook exome sequencing and RRBS sequencing for the normal, pRCC, IVC and MB tissues of a single patient. As shown above, using exome data as the control, we found the accuracy of SNP detection in real data to be lower than that in simulated data for Bowtie2, BSMAP and Bismark pipelines. This finding could be due to the greater complexity of real data. We also noticed that the Bismark pipeline performed best in our real data test in terms of the accuracy of SNP calling (Additional file 1: Table S5). When testing with simulated data, BSMAP illustrated a higher mapping rate whereas Bismark performed more accurately. When read length increased from 50 bp to 90 bp, the accuracy of BSMAP increased considerably, whereas Bismark showed an improved mapping rate (Additional file 1: Table S2). ASM detection has critical roles in analyses of methylation data. In our *in silico* experiments, BSMAP and Bismark pipelines performed similarly for 50 bp read PE data. For 90 bp read PE data, the Bismark pipeline showed higher accuracy whereas BSMAP showed better sensitivity. The Bowtie2 pipeline showed lower power to detect heterozygous SNPs using *in silico* data (Table 1). Thus, we kept both the pipelines and let users decide which to choose. To obtain higher accuracy, the Bismark pipeline is recommended. However, if one wants to cover as many SNPs as possible, the BSMAP pipeline should be chosen. For short reads (e.g., 50 bp), the Bismark pipeline could be a better choice, whereas the BSMAP pipeline works well for longer reads (e.g., 90 bp). Future work should be focused on combining their advantages and avoiding their disadvantages, thus improving sensitivity and accuracy. All pipelines performed well and did not show significant differences in methylation detection (Table S3 in Additional file 1).

SMAP runs in a UNIX/Linux shell. A graphical user interface has been implemented. SMAP is optimized for parallel computing platforms, including single multi-core computing nodes and clusters. For cluster computing environments, the current version of SMAP supports only job-management systems based on Sun Grid Engine. Future versions will add support for other job-management systems such as Simple Linux Utility for Resource Management.

## Availability and requirements

**Project Name:** SMAP: a streamlined methylation analysis pipeline for bisulfite sequencing

**Project home page:** https://github.com/gaosjlucky/SMAPdigger

**Operating system:** Linux

**Programming language:** Perl, R and Java

**Other requirements**: See documentation for a comprehensive list of optional dependencies.

**License:** GPL v3

## Availability of supporting data

SRA accession of data used in this paper is SRP058673. Example data for the pipeline testing are available from the GigaScience GigaDB database [37]. Source code of the pipeline is freely available at https://github.com/gaosjlucky/SMAPdigger.

## Additional files

Additional file 1: Figure S1. Coverage of CpG islands in the normal tissue of a patient with metastatic renal cell carcinoma. Figure S2. Coverage of CpG islands in primary renal cell carcinoma (pRCC) tissue of a patient with metastatic renal cell carcinoma. Figure S3. Coverage of CpG islands in local invasion of the vena cava (IVC) tissue of a patient with metastatic renal cell carcinoma. Figure S4. Coverage of CpG islands in distant metastasis to the brain (MB) tissue of a patient with metastatic renal cell carcinoma. Figure S5. Venn diagram showing how SNPs are shared in four real datasets. Figure S6. Venn diagram showing how SNPs are shared in BSMAP and Bismark pipelines *in silico*. Table S1. Comparison of analytical features of programs for evaluation of genome-wide methylation data. Table S2. Comparison of mapping performance between BSMAP and Bismark pipelines *in silico*. Table S3. Performance of methylation detection *in silico*. Table S4. Validation of DMR in primary renal cell carcinomas (pRCC) and normal tissues. Table S5. Comparison of the performance of BSMAP, Bismark and Bowtie2 pipelines with real data. Additional file 2:
**Documentation for SMAP with a full example.**

## Abbreviations

ASM: Allele-specific DNA methylation
DMC: Differentially methylated C sites
DMR: Differentially methylated regions
IVC: Local invasion of the vena cava
MB: Distant metastasis to brain tissues
pRCC: Primary renal cell carcinomas
RRBS: Reduced representation bisulfite sequencing
PE: Paired end
SE: Single end
