# Supplementary material for: SMAP: a streamlined methylation analysis pipeline for bisulfite sequencing
Source: Gigascience. 2015 Jul 1;4:29. doi: 10.1186/s13742-015-0070-9 (PMC4488126; doi:10.1186/s13742-015-0070-9)
Supplement: Additional file 1: Figure S1. — Coverage of CpG islands in the normal tissue of a patient with metastatic renal cell carcinoma. Figure S2. Coverage of CpG islands in primary renal cell carcinoma (pRCC) tissue of a patient with metastatic renal cell carcinoma. Figure S3. Coverage of CpG islands in local invasion of the vena cava (IVC) tissue of a patient with metastatic renal cell carcinoma. Figure S4. Coverage of CpG islands in distant metastasis to the brain (MB) tissue of a patient with metastatic renal cell carcinoma. Figure S5. Venn diagram showing how SNPs are shared in four real datasets. Figure S6. Venn diagram showing how SNPs are shared in BSMAP and Bismark pipelines in silico. Table S1. Comparison of analytical features of programs for evaluation of genome-wide methylation data. Table S2. Comparison of mapping performance between BSMAP and Bismark pipelines in silico. Table S3. Performance of methylation detection in silico. Table S4. Validation of DMR in primary renal cell carcinomas (pRCC) and normal tissues. Table S5. Comparison of the performance of BSMAP, Bismark and Bowtie2 pipelines with real data. [file 13742_2015_70_MOESM1_ESM.docx]

**Additional Materials**


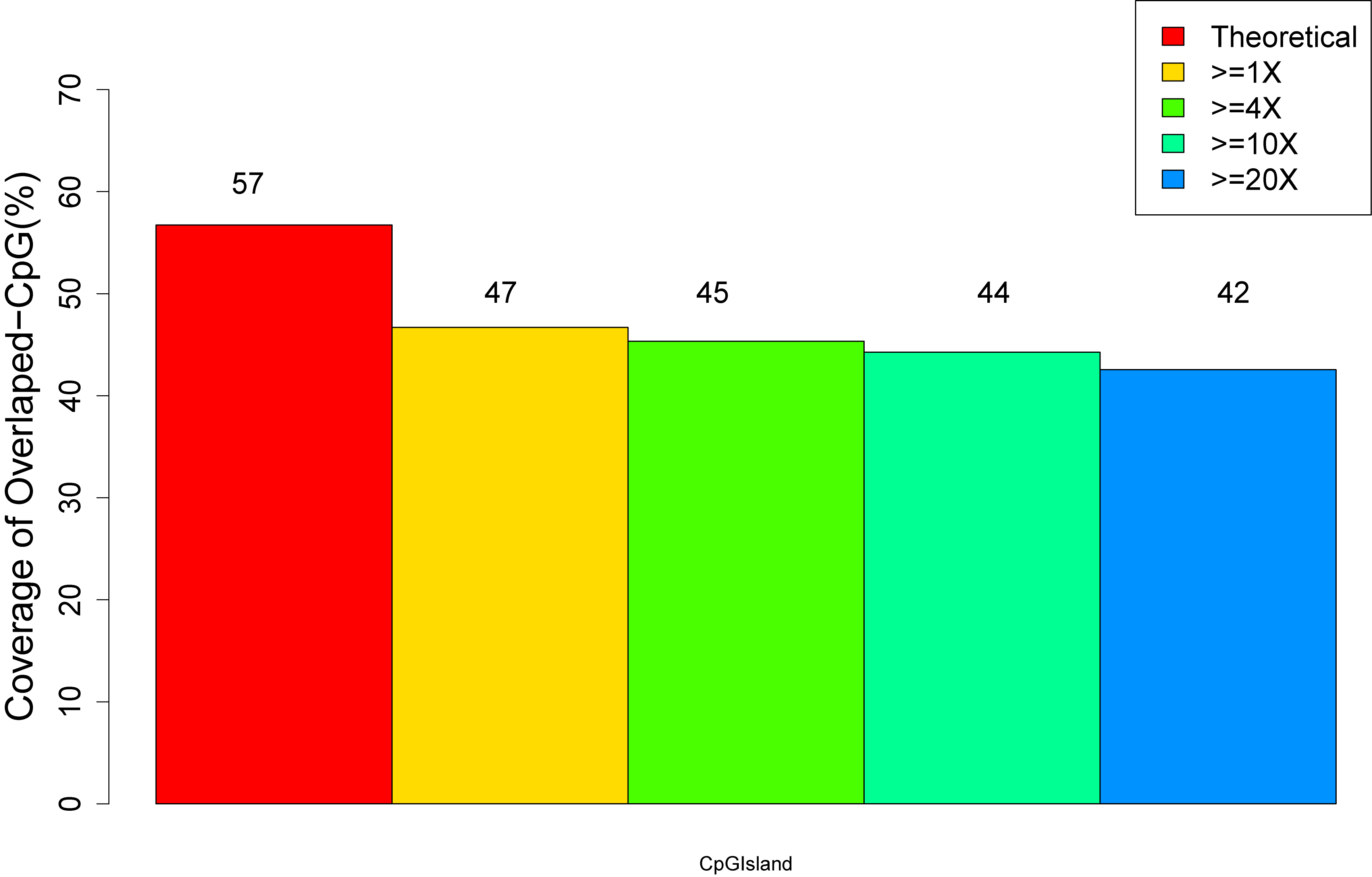


# Supplementary Figure 1. Coverage of CpG islands in the normal tissue of a patient with metastatic renal cell carcinoma.

**
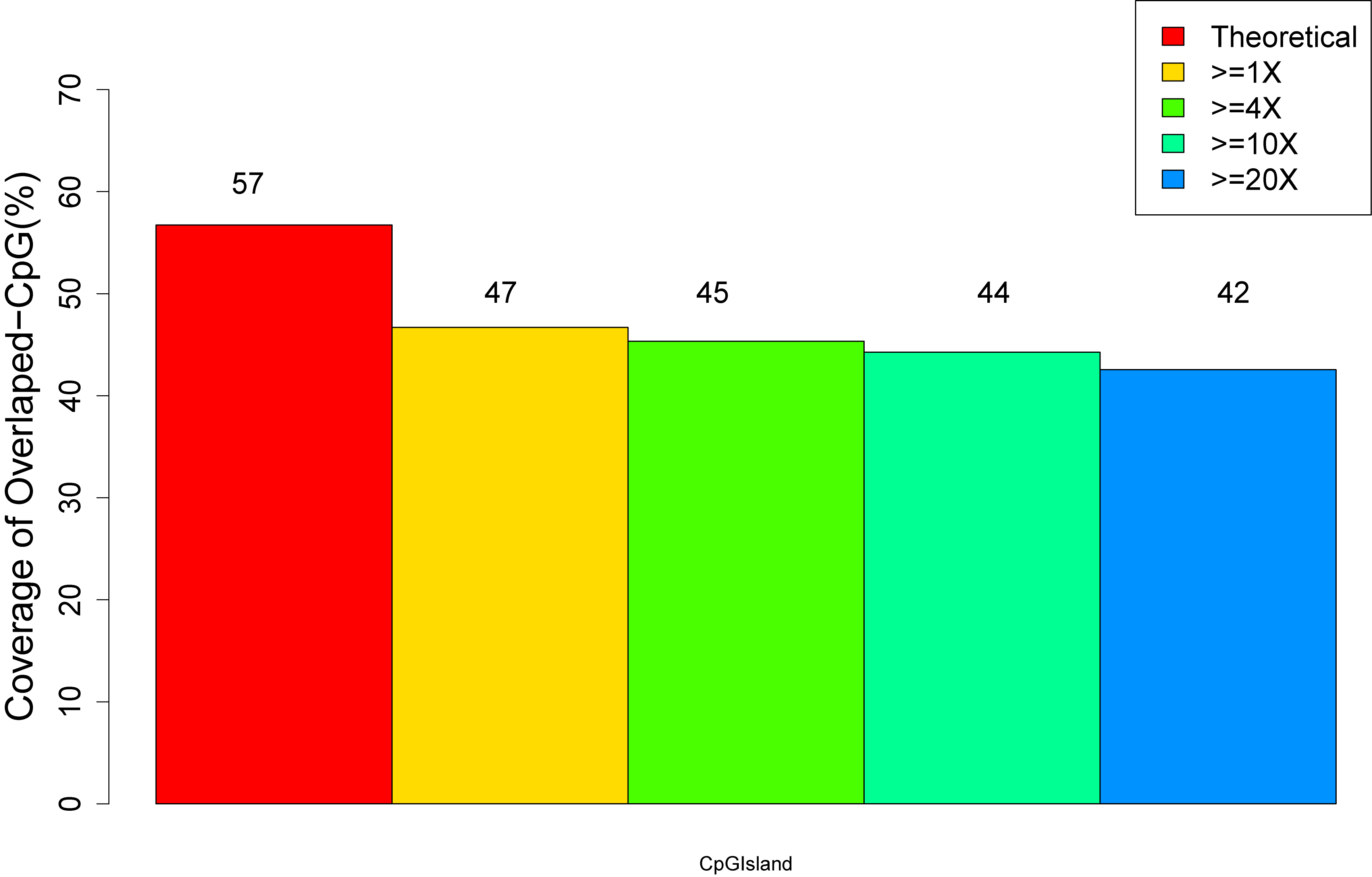
**

# Supplementary Figure 2. Coverage of CpG islands in primary renal cell carcinoma (pRCC) tissue of a patient with metastatic renal cell carcinoma.

**
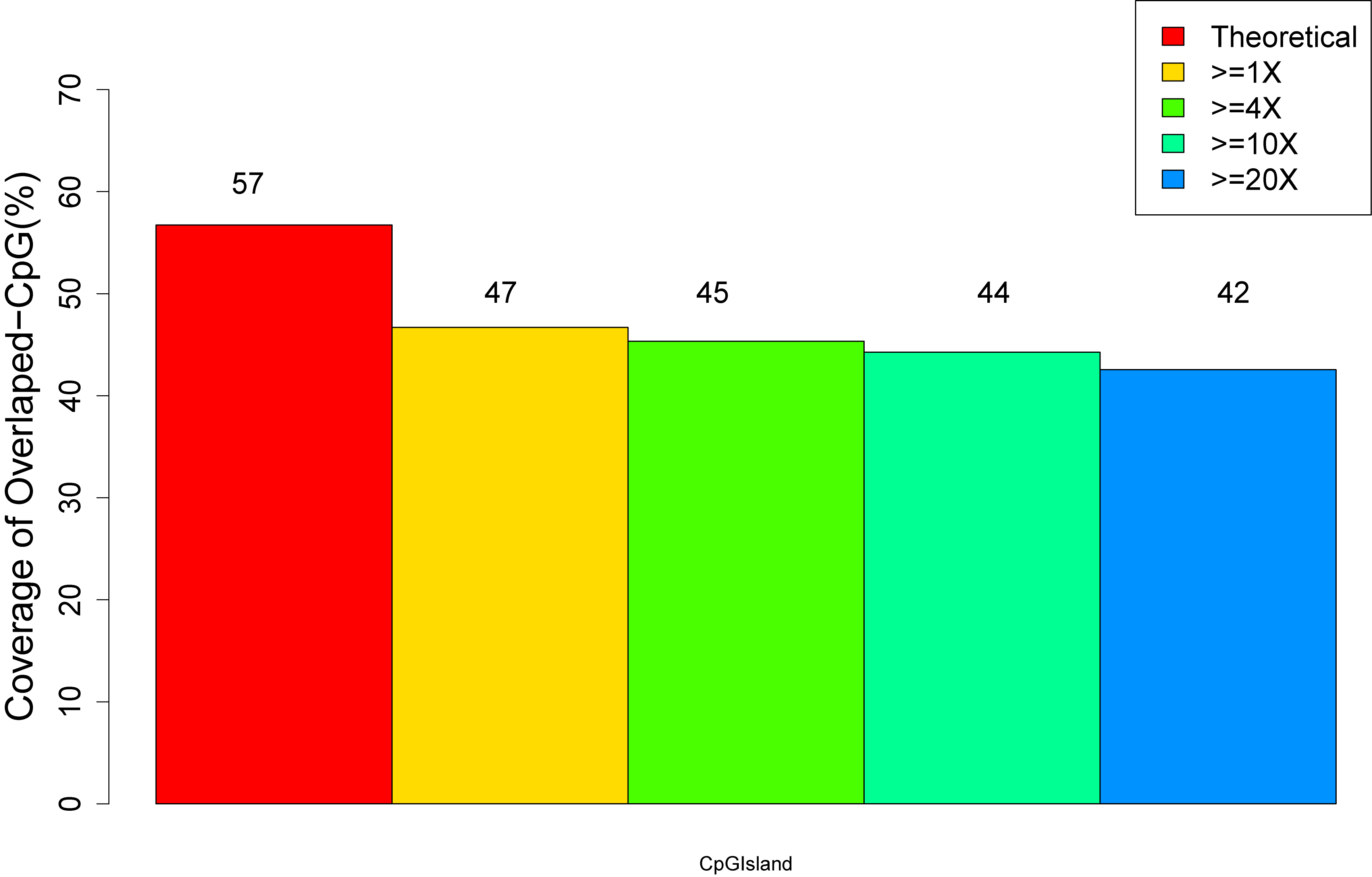
**

# Supplementary Figure 3. Coverage of CpG islands in local invasion of the vena cava (IVC) tissue of a patient with metastatic renal cell carcinoma.


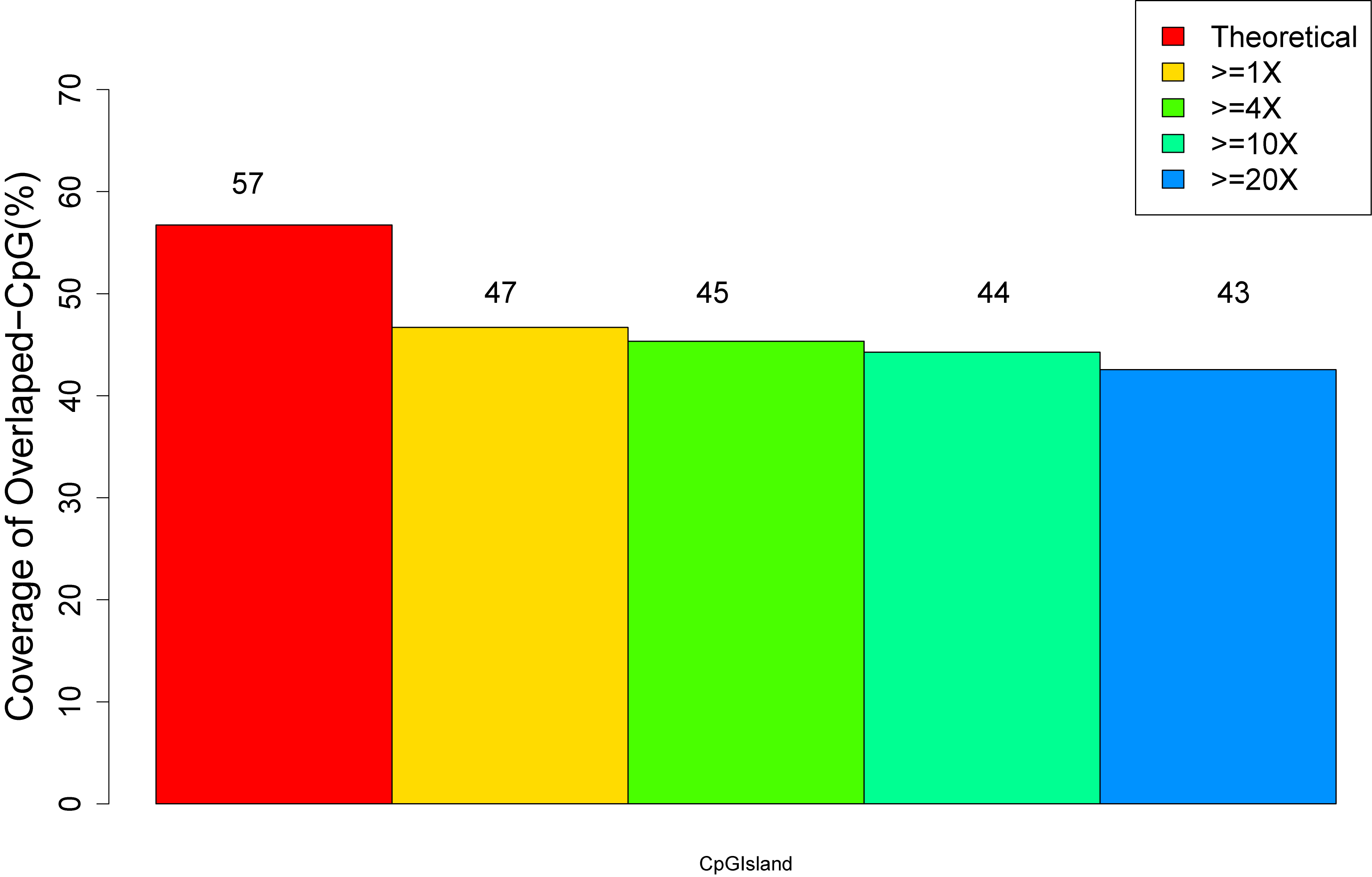


# Supplementary Figure 4. Coverage of CpG islands in distant metastasis to the brain (MB) tissue of a patient with metastatic renal cell carcinoma.


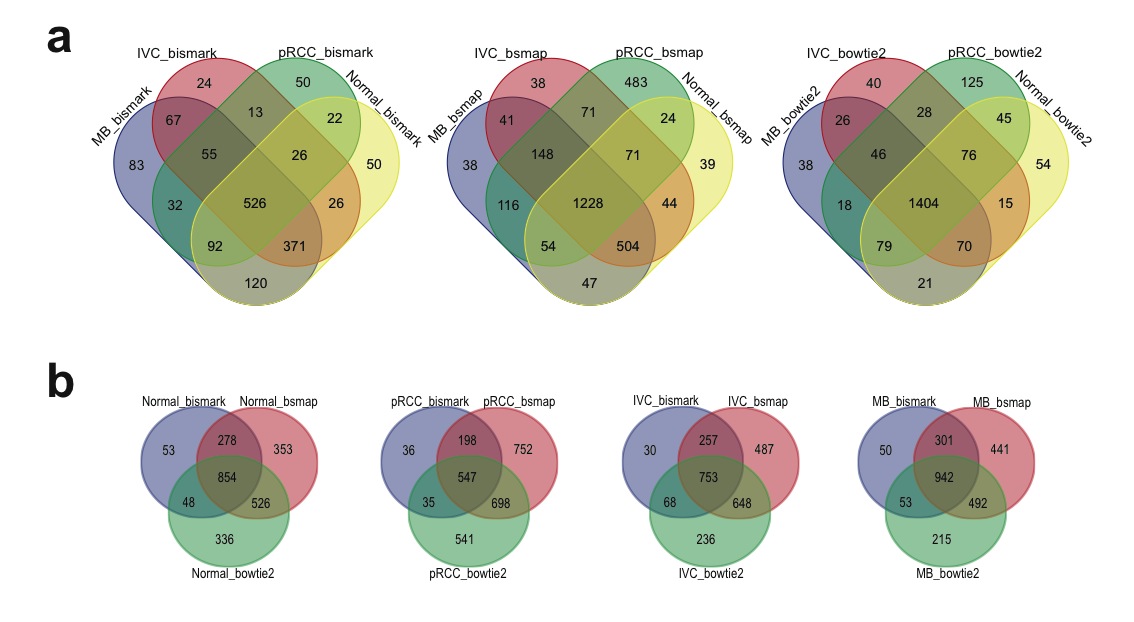


# Supplementary Figure 5. Venn diagram showing how SNPs are shared in four real datasets. (a) Shared numbers of correctly estimated SNPs in different tissues using Bowtie2, Bismark and BSMAP pipelines. (b) Shared numbers of correctly estimated SNPs detected by two pipelines in four tissues.


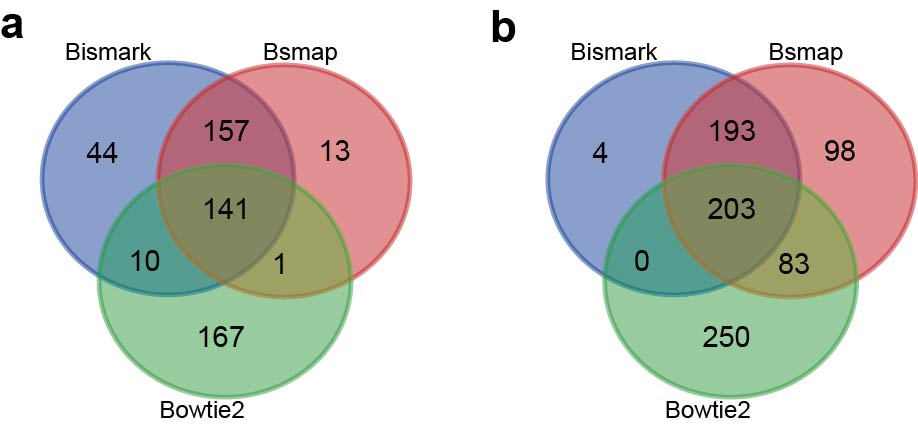


# Supplementary Figure 6. Venn diagram showing how SNPs are shared in BSMAP and Bismark pipelines. (a) PE50 data *in silico*. (b) PE90 data *in silico*.

# Supplementary Table 1. Comparison of analytical features of programs for evaluation of genome-wide methylation data.*

| **Software** | **Input file format(s)** | **Feature(s)** | **Output file format(s)** | **Program type** |
| --- | --- | --- | --- | --- |
| BIGpre [[1](#_ENREF_1)] | FASTQ | A | Tab-delimited text, R file (for plotting) | Command line (Perl) |
| BSeQC [[2](#_ENREF_2)] | SAM, BAM | A | SAM, BAM, PDF, text | Command line (Python) |
| Fastqc [[3](#_ENREF_3)] | FASTQ | A | HTML report | GUI (Java), Command line (Java) |
| HTQC [[4](#_ENREF_4)] | FASTQ | A | FASTQ, tab-delimited text | Command line (C++) |
| Toolkit [[5](#_ENREF_5)] | FASTQ | A | FASTQ, HTML report, tab-delimited text | Command line (R library) |
| PIQA [[6](#_ENREF_6), [7](#_ENREF_7)] | FASTQ | A | HTML report | Command line (R library) |
| QC-Chain | FASTQ | A | FASTQ | Command line (binary, C++) |
| SolexaQA [[7](#_ENREF_7)] | FASTQ | A | FASTQ, PDF, text | Command line (Perl, R library) |
| RUbioSeq [[8](#_ENREF_8)] | FASTQ | A, B, C | SAM/BAM, bed, wig, VCF | Command line (Perl) |
| WBSA [[9](#_ENREF_9)] | FASTQ | A, B , C, E, F, G | SAM, HTML report, tab-delimited text, genome browser | Web server |
| BRAT [[10](#_ENREF_10)] | FASTQ | B | Tab-delimited text (convertible to SAM) | Command line (C++) |
| BRAT-BW [[11](#_ENREF_11), [12](#_ENREF_12)] | FASTQ | B | Tab-delimited text (convertible to SAM) | Command line (C++) |
| BSMAP [[12](#_ENREF_12)] | FASTQ, BAM | B | SAM/BAM, BSP, text | Command line (C++) |
| BS-Seeker [[13](#_ENREF_13)] | FASTQ | B | SAM/BAM, tab-delimited text | Command line (Python) |
| GSNAP [[14](#_ENREF_14)] | FASTQ | B | SAM | Command line (C/Perl) |
| SOAP [[15](#_ENREF_15)] | FASTA | B | Tab-delimited text, SAM | Command line (C++) |
| BatMeth [[16](#_ENREF_16)] | FASTQ | B, C | Custom text format, PDF | Command line (C/C++) |
| Bismark [[17](#_ENREF_17)] | FASTQ | B, C | SAM, tab-delimited text | Command line (Perl) |
| BS-Seeker2 [[18](#_ENREF_18)] | FASTQ | B, C | SAM/BAM, tab-delimited text, wig | Command line (Python) |
| MethylCoder [[19](#_ENREF_19)] | FASTQ | B, C | SAM, tab-delimited text | Command line (Python) |
| MethyQA [[20](#_ENREF_20)] | FASTQ | B, C | Tab-delimited text | Command line (C++) |
| PASS-bis [[21](#_ENREF_21)] | FASTQ | B, C | Modified SAM | Command line (C++) |
| MethPipe [[22](#_ENREF_22)] | FASTQ, BAM, SAM | B, C, G, Ia | BAM, tab-delimited text | Command line (C++) |
| DMEAS [[20](#_ENREF_20)] | Bismark output (.bis) | C, D, E | Tab-delimited text, images | Command line (Perl) |
| CpG_MPs [[23](#_ENREF_23)] | Bismark output, text | C, D, E, G | Tab-delimited text, xls, images, genome browser | Java applet |
| GBSA [[24](#_ENREF_24)] | BSP, BS-Seeker (text) | C, E, F | Bedgraph, tab-delimited text, images, gene browser | GUI (Windows), Command line (Python) |
| MethylKit [[25](#_ENREF_25)] | Tab-delimited text | C, E, F, G | Bedgraph, tab-delimited text, images | Command line (R library) |
| UCSC Genome Bowser [[26](#_ENREF_26)] | Bismark ouput BAM, bedgraph, VCF, wig | E | Genome browser | Web server |
| VEP [[27](#_ENREF_27)] | Whitespace-delimited, VCF | F | Tab-delimited text, VCF | Web server, Command line (Perl) |
| Bis-SNP [[28](#_ENREF_28)] | BAM | C, H | Tab-delimited text, VCF | Java applet |
| DMAP [[29](#_ENREF_29)] | Bismark ouput | C，E，F，G | Tab-delimited text | Command line (C++) |
| SMAP | FASTQ | A, B, C, E, F, G, H, Ib, J | Tab-delimited text, VCF, images | GUI (Linux), Command line (Perl, R) |

*We integrated Table 1 in [[30](#_ENREF_30)] and added our work in this table.

A: QC; B: alignment; C: methylation scoring; D: quantitative score assessment; E: visualization; F: annotation; G: determination of differential DNA methylation; H: SNP detection; Ia: ASM analysis (using Amrfinder); Ib: ASM analysis (based on heterozygous SNPs); J: PE overlap treatment.

# Supplementary Table 2. The comparison of mapping performance between BSMAP and Bismark *in silico*.

|  | **# total reads** | **BSMAP** | | | |  | **Bismark** | | | |
| --- | --- | --- | --- | --- | --- | --- | --- | --- | --- | --- |
|  |  | **MR** | **AMR** | **FPR** | **FNR** |  | **MR** | **AMR** | **FPR** | **FNR** |
| **PE50** | 276760 | 276538 | 219886 | 0.21 | 0 |  | 238608 | 233541 | 0.02 | 0.14 |
| **PE60** | 276760 | 276640 | 239210 | 0.13 | 0 |  | 248555 | 246072 | 0.01 | 0.1 |
| **PE70** | 276760 | 276760 | 250853 | 0.09 | 0 |  | 253610 | 251645 | 0.01 | 0.08 |
| **PE80** | 276760 | 276760 | 258141 | 0.07 | 0 |  | 254470 | 252880 | 0.01 | 0.08 |
| **PE90** | 276760 | 276760 | 262228 | 0.05 | 0 |  | 250525 | 249473 | 0 | 0.09 |

Abbreviations: MR: the number of mapped reads; AMR: the number of accurately mapped reads; FPR: false-positive rate; FNR: false-negative rate.

# Supplementary Table 3. The performance of methylation detection *in silico*.

| **Start:End** | **Theoretical values** | |  |  | **Estimated values** | | | | | | | |
| --- | --- | --- | --- | --- | --- | --- | --- | --- | --- | --- | --- | --- |
|  | **Rm (C)** | **Rm (N)** |  |  | **Rm (C50T)** | **Rm (N50T)** | **Rm (C90T)** | **Rm (N90T)** | **Rm (C90M)** | **Rm (N50M)** | **Rm (C90M)** | **Rm (N90M)** |
| 10840:7173856 | 0.1 | 0.1 |  |  | 0.11 | 0.10 | 0.11 | 0.09 | 0.12 | 0.11 | 0.11 | 0.10 |
| 7174993:12377197 | 1 | 0 |  |  | 1.00 | 0.00 | 1.00 | 0.00 | 1.00 | 0.00 | 1.00 | 0.00 |
| 12377258:19791219 | 0 | 1 |  |  | 0.00 | 1.00 | 0.00 | 1.00 | 0.00 | 1.00 | 0.00 | 1.00 |
| 19793485:30349690 | 0.5 | 0.1 |  |  | 0.50 | 0.10 | 0.50 | 0.10 | 0.52 | 0.11 | 0.51 | 0.10 |
| 30350542:43963808 | 0.1 | 0.5 |  |  | 0.09 | 0.49 | 0.10 | 0.49 | 0.09 | 0.50 | 0.10 | 0.50 |
| 43969498:49866894 | 0.7 | 0.3 |  |  | 0.69 | 0.30 | 0.70 | 0.30 | 0.70 | 0.31 | 0.70 | 0.30 |
| 49972394:59634023 | 0.3 | 0.7 |  |  | 0.30 | 0.70 | 0.31 | 0.71 | 0.31 | 0.71 | 0.31 | 0.72 |
| 59647120:72163528 | 0.1 | 0.5 |  |  | 0.10 | 0.49 | 0.10 | 0.50 | 0.11 | 0.52 | 0.10 | 0.51 |
| 72164007:76469542 | 0.5 | 0.1 |  |  | 0.50 | 0.09 | 0.50 | 0.10 | 0.51 | 0.10 | 0.50 | 0.10 |
| 76473832:77971764 | 0.5 | 0.5 |  |  | 0.50 | 0.50 | 0.50 | 0.50 | 0.50 | 0.50 | 0.49 | 0.50 |

Abbreviations: Rm: methylation rate; C: Cancer; N: Normal; 50: PE50; 90: PE90; T: Bismark/Bowtie2; M: BSMAP.

# Supplementary Table 4. The validation of DMR in primary renal cell carcinomas (pRCC) and Normal tissues.

| **Gene** | **Sample** | **#All CpGs** | **#uCpG** | **#mCpG** | **Rm (%)** | **p value** | **Validated?** | **Target region** |
| --- | --- | --- | --- | --- | --- | --- | --- | --- |
| SLC5A7 | C | 484 | 358 | 126 | 26.0 | 2.15E-12 | Yes | chr2:107969359:107969444 |
|  | N | 588 | 545 | 43 | 7.3 |  |  |  |
| CD01 | C | 229 | 111 | 118 | 51.5 | 1.82E-13 | Yes | chr5:115180214:115180511 |
|  | N | 185 | 174 | 11 | 5.9 |  |  |  |
| CRMP1 | C | 407 | 271 | 136 | 33.4 | 1.86E-05 | Yes | chr4:5943104:5943228 |
|  | N | 134 | 122 | 12 | 9.0 |  |  |  |
| ALX1 | C | 59 | 31 | 28 | 47.5 | 0.003903 | T | chr12:84197456:84197648 |
|  | N | 123 | 101 | 22 | 17.9 |  |  |  |
| TBC1D1 | C | 128 | 51 | 77 | 60.2 | 8.74E-09 | Yes | chr4:37568194:37568432 |
|  | N | 304 | 247 | 57 | 18.8 |  |  |  |
| MAL | C | 308 | 228 | 80 | 26.0 | 4.54E-05 | Yes | chr2:95054506:95054901 |
|  | N | 308 | 276 | 32 | 10.5 |  |  |  |
| DES | C | 199 | 134 | 65 | 32.7 | 3.75E-04 | Yes | chr2:219991376:219991474 |
|  | N | 299 | 254 | 45 | 15.1 |  |  |  |
| DDX25 | C | 221 | 132 | 89 | 40.3 | 6.17E-10 | Yes | chr11:125279592:125279697 |
|  | N | 419 | 367 | 52 | 12.4 |  |  |  |
| USP44 | C | 605 | 443 | 162 | 26.8 | 8.75E-06 | Yes | chr12:94466217:94466921 |
|  | N | 203 | 186 | 17 | 8.4 |  |  |  |
| PRIMA1 | C | 352 | 297 | 55 | 15.6 | 1.20E-08 | Yes | chr14:93323531:93323906 |
|  | N | 416 | 245 | 171 | 41.1 |  |  |  |
| ZNF177 | C | 108 | 78 | 30 | 27.8 | 0.0295 | Yes | chr19:9334684:9334856 |
|  | N | 119 | 104 | 15 | 12.6 |  |  |  |
| C9orf75 | C | 348 | 189 | 159 | 45.7 | 2.32E-10 | Yes | chr9:139213596:139214223 |
|  | N | 348 | 295 | 53 | 15.2 |  |  |  |

Abbreviations: C: Cancer, N: Normal; Rm: methylation rate; uCpG: Unmethylated CpGs; mCpG: Methylated CpGs.

# Supplementary Table 5. Comparison of the performance of BSMAP, Bismark and Bowtie2 pipelines with real data.

|  | **BSMAP + Bis-SNP** | | | |  | **Bismark + Bis-SNP** | | | |  | **Bowtie2 + Bcftools** | | | |
| --- | --- | --- | --- | --- | --- | --- | --- | --- | --- | --- | --- | --- | --- | --- |
|  | **MB** | **IVC** | **pRCC** | **Normal** |  | **MB** | **IVC** | **pRCC** | **Normal** |  | **MB** | **IVC** | **pRCC** | **Normal** |
| # Exon SNPs* | 2966 | 2995 | 3401 | 2873 |  | 2827 | 2839 | 2931 | 2786 |  | 2375 | 2502 | 2528 | 2513 |
| # Target SNPs | 4355 | 3906 | 3308 | 3483 |  | 1404 | 1151 | 844 | 1268 |  | 3258 | 3353 | 2924 | 3137 |
| # SNPs validated | 2176 | 2145 | 2195 | 2011 |  | 1346 | 1108 | 816 | 1233 |  | 1702 | 1705 | 1821 | 1764 |
| FPR | 0.5 | 0.45 | 0.34 | 0.42 |  | 0.04 | 0.04 | 0.03 | 0.03 |  | 0.48 | 0.49 | 0.38 | 0.44 |
| FNR | 0.27 | 0.28 | 0.35 | 0.3 |  | 0.52 | 0.61 | 0.72 | 0.56 |  | 0.28 | 0.32 | 0.28 | 0.30 |

^*^Whole exome-sequenced data were used to validate overlapped target SNPs in RRBS regions and exome target regions. Abbreviations: FPR: false-positive rate; FNR: false-negative rate; Target: overlap of exome target regions and RRBS target regions. Normal: normal tissue; pRCC: primary renal cell carcinomas; IVC: local invasion of the vena cava; MB: distant metastasis to the brain; Exon SNPs: SNPs detected in exon target regions; Target SNPs: SNPs detected in target regions.

# References

1. Zhang T, Luo Y, Liu K, Pan L, Zhang B, Yu J, Hu S. BIGpre: a quality assessment package for next-generation sequencing data. Genomics, Proteomics Bioinformatics. 2011;9:238-44.

2. Lin X, Sun D, Rodriguez B, Zhao Q, Sun H, Zhang Y, Li W. BSeQC: quality control of bisulfite sequencing experiments. Bioinformatics. 2013; 29:3227-9.

3. S. A. FastQC: a quality control tool for high throughput sequence data. <http://wwwbioinformaticsbabrahamacuk/projects/fastqc/> (Accessed 14 April 2014).

4. Yang X, Liu D, Liu F, Wu J, Zou J, Xiao X, Zhao F, Zhu B. HTQC: a fast quality control toolkit for Illumina sequencing data. BMC Bioinform. 2013;14:33.

5. Patel RK, Jain M. NGS QC Toolkit: a toolkit for quality control of next generation sequencing data. PloS One*.* 2012;7:e30619.

6. Martinez-Alcantara A, Ballesteros E, Feng C, Rojas M, Koshinsky H, Fofanov VY, Havlak P, Fofanov Y. PIQA: pipeline for Illumina G1 genome analyzer data quality assessment. Bioinformatics. 2009;25:2438-9.

7. Cox MP, Peterson DA, Biggs PJ. SolexaQA: At-a-glance quality assessment of Illumina second-generation sequencing data. BMC Bioinformatics. 2010;11:485.

8. Rubio-Camarillo M, Gomez-Lopez G, Fernandez JM, Valencia A, Pisano DG. RUbioSeq: a suite of parallelized pipelines to automate exome variation and bisulfite-seq analyses. Bioinformatics. 2013;29:1687-9.

9. Liang F, Tang B, Wang Y, Wang J, Yu C, Chen X, Zhu J, Yan J, Zhao W, Li R. WBSA: web service for bisulfite sequencing data analysis. PloS One*.*2014;9:e86707.

10. Harris EY, Ponts N, Levchuk A, Roch KL, Lonardi S. BRAT: bisulfite-treated reads analysis tool. Bioinformatics*.* 2010;26:572-3.

11. Harris EY, Ponts N, Le Roch KG, Lonardi S. BRAT-BW: efficient and accurate mapping of bisulfite-treated reads. Bioinformatics. 2012;28:1795-6.

12. Xi Y, Li W. BSMAP: whole genome bisulfite sequence MAPping program. BMC Bioinform 2009;10:232.

13. Chen PY, Cokus SJ, Pellegrini M. BS Seeker: precise mapping for bisulfite sequencing. *BMC Bioinform* 2010;11:203.

14. Wu TD, Nacu S. Fast and SNP-tolerant detection of complex variants and splicing in short reads. Bioinformatics. 2010;26:873-81.

15. Li R, Li Y, Kristiansen K, Wang J. SOAP: short oligonucleotide alignment program. Bioinformatics*.* 2008;24:713-14.

16. Lim JQ, Tennakoon C, Li G, Wong E, Ruan Y, Wei CL, Sung WK. BatMeth: improved mapper for bisulfite sequencing reads on DNA methylation. Genome Biol. 2012;13:R82.

17. Krueger F, Andrews SR. Bismark: a flexible aligner and methylation caller for Bisulfite-Seq applications. Bioinformatics*.* 2011;27(11):1571-2.

18. Guo W, Fiziev P, Yan W, Cokus S, Sun X, Zhang MQ, Chen PY, Pellegrini M. BS-Seeker2: a versatile aligning pipeline for bisulfite sequencing data. BMC Genomics.2013;14:774.

19. Pedersen B, Hsieh TF, Ibarra C, Fischer RL. MethylCoder: software pipeline for bisulfite-treated sequences. Bioinformatics. 2011;27:2435-6.

20. He J, Sun X, Shao X, Liang L, Xie H. DMEAS: DNA methylation entropy analysis software. Bioinformatics. 2013;29:2044-5.

21. Campagna D, Telatin A, Forcato C, Vitulo N, Valle G. PASS-bis: a bisulfite aligner suitable for whole methylome analysis of Illumina and SOLiD reads. Bioinformatics. 2013;29:268-270.

22. Song Q, Decato B, Hong EE, Zhou M, Fang F, Qu J, Garvin T, Kessler M, Zhou J, Smith AD. A reference methylome database and analysis pipeline to facilitate integrative and comparative epigenomics. *PloS One.* 2013;8:e81148.

23. Su J, Yan H, Wei Y, Liu H, Liu H, Wang F, Lv J, Wu Q, Zhang Y. CpG_MPs: identification of CpG methylation patterns of genomic regions from high-throughput bisulfite sequencing data. Nucl Acids Res. 2013;41:e4.

24. Benoukraf T, Wongphayak S, Hadi LH, Wu M, Soong R. GBSA: a comprehensive software for analysing whole genome bisulfite sequencing data. Nucl Acids Res. 2013;41:e55.

25. Akalin A, Kormaksson M, Li S, Garrett-Bakelman FE, Figueroa ME, Melnick A, Mason CE. methylKit: a comprehensive R package for the analysis of genome-wide DNA methylation profiles. Genome Biol. 2012;13:R87.

26. Meyer LR, Zweig AS, Hinrichs AS, *et al*. The UCSC Genome Browser database: extensions and updates 2013. Nucl Acids Res. 2013;41:D64-D69.

27. McLaren W, Pritchard B, Rios D, Chen Y, Flicek P, Cunningham F. Deriving the consequences of genomic variants with the Ensembl API and SNP Effect Predictor. Bioinformatics. 2010;26:2069-70.

28. Liu Y, Siegmund KD, Laird PW, Berman BP. Bis-SNP: Combined DNA methylation and SNP calling for Bisulfite-seq data. Genome Biol. 2012;13:R61.

29. Stockwell PA, Chatterjee A, Rodger EJ, Morison IM. DMAP: differential methylation analysis package for RRBS and WGBS data. Bioinformatics. 2014; doi: 10.1093/bioinformatics/btu126.

30. Adusumalli S, Mohd Omar MF, Soong R, Benoukraf T. Methodological aspects of whole-genome bisulfite sequencing analysis. *Brief Bioinform.* 2014; doi: 10.1093/bib/bbu016.
