# Supplementary material for: SMAP: a streamlined methylation analysis pipeline for bisulfite sequencing
Source: Gigascience. 2015 Jul 1;4:29. doi: 10.1186/s13742-015-0070-9 (PMC4488126; doi:10.1186/s13742-015-0070-9)
Supplement: Additional file 2: — Documentation for SMAP with a full example. [file 13742_2015_70_MOESM2_ESM.docx]

**Documentation for SMAP**

28 January 2015

Table of Contents

[1. Introduction 2](#_Toc413852986)

[2. System requirement 2](#_Toc413852987)

[3. Getting started 2](#_Toc413852988)

[3.1 Download of SMAP 2](#_Toc413852989)

[3.2 Installation of SMAP 3](#_Toc413852990)

[3.3 Usage 3](#_Toc413852991)

[4. Input file (configuration file) 3](#_Toc413852992)

[5. Output files 4](#_Toc413852993)

[5.1 QC output 4](#_Toc413852994)

[5.2 Single C’s methylation information 5](#_Toc413852995)

[5.3 DMR output 5](#_Toc413852996)

[5.4 SNP output 6](#_Toc413852997)

[5.5 ASM output 6](#_Toc413852998)

# Introduction

Streamlined methylation analysis pipeline (SMAP) is a flexible pipeline to streamline bisulfite sequencing (Bis-seq) and reduced representation bisulfite sequencing (RRBS) data processing of DNA methylation in multiple samples. Main features of SMAP include: (i) handling of single- and/or paired-end (SE/PE) diverse bisulfite sequencing data with a reduced false-positive rate in differentially methylated regions; (ii) detection of allele-specific methylation events with improved algorithms; (iii) built-in pipeline for detection of novel single nucleotide polymorphisms (SNPs); (iv) support of user-defined multiple restriction enzymes; (v) conduction of a one-step operation in all methylation analyses.

# System requirement

To run SMAP in command-line mode (without GUI), users need a 64-bit Linux system. If computational cluster use is desired, the job-management system Sun Grid Engine (SGE) should be installed on the cluster for the current version. To run SMAP with GUI, users need a 64-bit Linux system with ≥4 GB memory and installation of JAVA 1.7 or newer. Currently, GUI mode does not support job management.

# Getting started

## 3.1 Download of SMAP

SMAP package SMAPdigger-1.0.tar.gz can be downloaded from <https://github.com/gaosjlucky/SMAPdigger/releases>.

## 3.2 Installation of SMAP

1. Save SMAPdigger-1.0.tar.gz and extract it with command "tar vxzf SMAPdigger-1.0.tar.gz".

2. Execute command "chmod +x ./*.sh".

3. Execute command "./Software_Setup.sh".

## 3.3 Usage

To run SMAP in command-line mode, please follow the instructions in readme.txt in the downloaded package.

To run SMAP in GUI mode, double-click file smapDigger.jar or run command “java –jar smapDigger.jar”. Then, select the configuration file and output path. Click button “Set workspace configuration” to create scripts. Finally, click button “Run All” to run all steps or click one of the four buttons below to run one of the steps. Running status could be observed in the text view below.


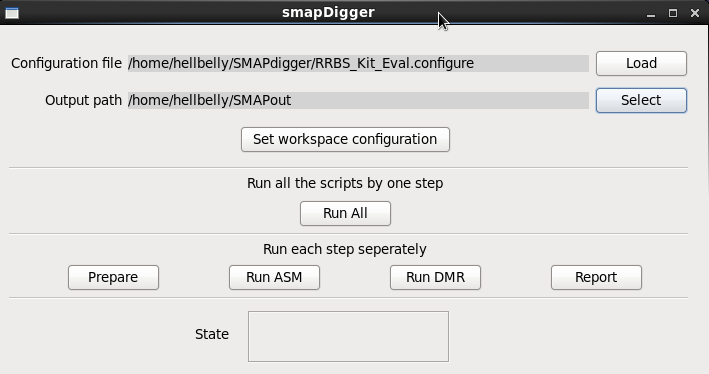


# Input file (configuration file)

Below is an example of a configuration file that comes with the package. A line beginning with a hash key “#” is a comment. Other lines follow the format of “key = value”.

#Reion, blank separated

Region = 40 220

#SGE switch : 0 - without SGE; 1 - with SGE

SGEsel = 0

#Breakpoint switch : 0 - without Breakpoint; 1 - with Breakpoint

BPTsel = 1

#qsub command arguments "-p" (project)

Project = HUMcccR

#qsub command arguments "-q" (queue)

Queue = bc.q

# Select software for alignment. bowtie2 / bsmap

# Alignsoft = /usr/bin/bsmap -z 64 -p 12 -s 12 -v 10 -q 2

Alignsoft = /usr/bin/bowtie2 --phred64 -p 8

# Absolute full path for java and R

Javasoft = /usr/bin/java

Rscript = /usr/bin/R

# Sample directory

SplDir = ./data/sample

# Adapter file name

Adapter = adapter.fa

# Sample follows '=' are blank separated

# name of tissue, Normal or Tumour, SE or PE, name of library, name of FlowCell, file name of read1, file name of read2, platform, target region.

Sample = PE90 Normal PE HUMkfoHABDDAAPEI-8 FCC076MACXX_L1 pe90normal.1.fq pe90normal.2.fq illumina 0 800

Sample = PE90 Cancer PE HUMkfoHADDDAAPEI-2 FCC01P5ACXX_L8 pe90tumor.1.fq pe90tumor.2.fq illumina 0 800

Sample = PE50 Normal PE HUMkfoHABDDAAPEI-8 FCC076MACXX_L1 pe50normal.1.fq pe50normal.2.fq illumina 0 800

Sample = PE50 Cancer PE HUMkfoHADDDAAPEI-2 FCC01P5ACXX_L8 pe50tumor.1.fq pe50tumor.2.fq illumina 0 800

# Output files

The outputs of SMAP are shown in this section. The outputs were created using the four tissue data (ccRCC) mentioned in the main text, which could be downloaded from the ftp site <ftp://public.genomics.org.cn/BGI/SMAP/> .

## QC output

Shown below is the QC output example of the four tissues from the patient with metastatic renal cell carcinoma mentioned in main text.

|  | K1:Normal | K1:pRCC | K1:MVC | K1:MB |
| --- | --- | --- | --- | --- |
| # Total base(MB) | 14771.79 | 16053.26 | 14067.79 | 13266.94 |
| # Total read(M reads) | 164.2 | 178.52 | 156.42 | 147.47 |
| # Clean base(MB) | 12594.43 | 14900.02 | 12235.82 | 11062.03 |
| # Clean read(M reads) | 162.3 | 176.84 | 154.88 | 145.81 |
| # Mapped reads in enzyme target region(M reads) | 88.98 | 98.07 | 75.56 | 83.42 |
| Rate of mapping reads in enzyme target regions | 0.97 | 0.84 | 0.93 | 0.98 |
| # Total enzyme 40-220 target regions | 649812 | 649812 | 649812 | 649812 |
| # Covered enzyme 40-220 target regions | 523522 | 523984 | 523456 | 509322 |
| Rate of covered enzyme 40-220 target regions | 0.81 | 0.81 | 0.81 | 0.78 |

Abbreviations: Normal: Normal tissue; pRCC: primary renal cell carcinomas; IVC: local invasion of the vena cava; MB: distant metastasis to the brain

## Information on methylation of single C’s

Shown below is part of the output example regarding information for methylation of a single C.

| chr21 | 9437434 | - | CHG | GGC | 1 | 0 | 86 | 87 |
| --- | --- | --- | --- | --- | --- | --- | --- | --- |
| chr21 | 9437436 | - | CHH | GTG | 1 | 1 | 85 | 5 |

…

Meanings of the fields of the single C’s methylation information output are:

1. Chromosome

2. Coordinate

3. Strand: ‘+’ or ‘-’

4. Base type: CG, CHG or CHH

5. Reference base type

6. Copy number: number of repeats of flanking regions in the genome

7. Number of methylated reads

8. Number of unmethylated reads

9. The cutoff number of reads with a p-value smaller than the cutoff (default, 0.01): Only if the number of methylated reads is larger than this number, then this position is defined as a methylated site.

## DMR output

Part of the DMR output of the example is shown below.

| chr1 | 1334718 | 1336717 | CCNL2:- | 82/82/82/90 | 0.00 | 0.00 | 3.66E-02 | 6.47E-02 |
| --- | --- | --- | --- | --- | --- | --- | --- | --- |
| chr1 | 1342693 | 1344692 | MRPL20:- | 149/149/149/152 | 0.08 | 0.10 | 9.43E-07 | 9.48E-01 |

…

Meanings of the fields of DMR output are:

1. Chromosome

2. Start position of a DMR

3. End position of a DMR

4. Annotation: Strand. Gene symbol of the gene whose promoter or CpG island overlaps with the DMR.

5. overlap_num/methy_in_normal/methy_in_case/total_number

6. case_methy_ratio

7. control_methy_ratio

8. p-value of the chi-square test

9. p-value of the Student’s *t*-test

## SNP output

For the current version of SMAP, we use Bis-SNP or Bcftools to detect SNPs. The format of SNP is variant call format (VCF). Please check <http://www.1000genomes.org/wiki/analysis/variant%20call%20format/vcf-variant-call-format-version-41> for details of this format.

## ASM output

Below is part of the ASM output of the example.

| chr21_11128701 | 11128671 | A | G | 128 | 56 | 34 | 2 | 0.003 |
| --- | --- | --- | --- | --- | --- | --- | --- | --- |
| chr21_11128701 | 11128709 | A | G | 148 | 36 | 36 | 0 | 0.008 |

…

Meanings of the fields of ASM output are:

1. SNP position (Chromosome_coordinate)

2. ASM position

3. Reference

4. Variance

5. Number of methylated reads in the reference haplotype

6. Number of unmethylated reads in the reference haplotype

7. Number of methylated reads in the variant haplotype

8. Number of unmethylated reads in the variant haplotype

9. p-value of the chi-square test
